# MSCDNet-based multi-class classification of skin cancer using dermoscopy images

**DOI:** 10.7717/peerj-cs.1520

**Published:** 2023-08-29

**Authors:** Vankayalapati Radhika, B. Sai Chandana

**Affiliations:** School of Computer Science Engineering, VIT-AP University, Amaravathi, India

**Keywords:** Early detection, Deep learning, Performance results, Semantic segmentation, Relevant features

## Abstract

**Background:**

Skin cancer is a life-threatening disease, and early detection of skin cancer improves the chances of recovery. Skin cancer detection based on deep learning algorithms has recently grown popular. In this research, a new deep learning-based network model for the multiple skin cancer classification including melanoma, benign keratosis, melanocytic nevi, and basal cell carcinoma is presented. We propose an automatic Multi-class Skin Cancer Detection Network (MSCD-Net) model in this research.

**Methods:**

The study proposes an efficient semantic segmentation deep learning model “DenseUNet” for skin lesion segmentation. The semantic skin lesions are segmented by using the DenseUNet model with a substantially deeper network and fewer trainable parameters. Some of the most relevant features are selected using Binary Dragonfly Algorithm (BDA). SqueezeNet-based classification can be made in the selected features.

**Results:**

The performance of the proposed model is evaluated using the ISIC 2019 dataset. The DenseNet connections and UNet links are used by the proposed DenseUNet segmentation model, which produces low-level features and provides better segmentation results. The performance results of the proposed MSCD-Net model are superior to previous research in terms of effectiveness and efficiency on the standard ISIC 2019 dataset.

## Introduction

Uncontrolled, abnormal skin cell development causes skin cancer, which produces malignant tumors (Hekler et al., 2019). Skin cancer develops due to a DNA mutation; UV rays damage these cells if exposed to them. This mutation interferes with the normal proliferation of skin cells (Harangi, 2018). The World Health Organization says thirty percent of all cancer cases globally are skin cancer (Fujisawa, Inoue & Nakamura, 2019). People worldwide are impacted by the public health problem of skin cancer (Tan et al., 2018; Ashraf et al., 2020).

A standard method to find skin cancer is dermoscopy. The dermatologist finds it difficult to precisely diagnose different skin malignancies because their initial appearance is similar (Zhang et al., 2020). When using dermoscopic images to determine skin cancer, dermatologists typically have an accuracy rate between 60% and 80% (Vijayalakshmi, 2019). More than 60% accuracy is reportedly stated for a dermatologist with three to five years of experience (Kumar et al., 2020). A dermatologist with less experience performs less accurately (Jinnai et al., 2020). Moreover, the accuracy rate of 80% is represented by dermatologists who have ten years of experience. Additionally, the accuracy of skin cancer detection is impaired by untrained dermatologists. The essential need for dermoscopy is extensive training (Ameri, 2020). Dermoscopic images can diagnose a wide range of skin cancers (Sreelatha, Subramanyam & Prasad, 2019). But the two primary categories of skin disease are melanocytic and nonmelanocytic (Dorj et al., 2018). Melanoma and melanocytic nevi are both parts of the melanotic. In contrast, nonmelanocytic type skin diseases are dermatofibroma (DF), benign keratosis lesions (BKL), vascular (VASC), squamous cell carcinoma (SCC), and basal cell carcinoma (BCC) (Amin et al., 2020).

One well-known type of skin cancer is melanoma, among several skin lesions, which form of skin condition is often the most hazardous (Kaur et al., 2022). Melanoma is one of the fastest-spreading skin malignancies (Nahata & Singh, 2020). Using automatic computer-aided methods, accurate skin lesion classification is advantageous for preserving human life (Adegun & Viriri, 2019).

Melanoma is challenging to identify from dermoscopy images because of three major problems. First of all, although skin lesions fall under many classes, it is difficult to skin cancer classification due to how similar their size, texture, color, and type (Ajmal et al., 2022). The biological factors include illumination, veins, and hair. These difficulties for dermatologists are solved by establishing a computer aided design (CAD) system (Banerjee et al., 2020). For the evaluation of dermoscopic images, the CAD system also uses machine learning and image processing (Khan et al., 2022b). More data is made accessible for computer-aided systems, which improves the diagnosis (Jojoa Acosta et al., 2021). As a result, the development of artificial intelligence (AI) based detection system is the main objective of researchers for early detection and classification of multiple skin cancer types (Li & Shen, 2018). Additionally, deep learning and machine learning techniques are used by clinicians to identify skin lesions early, which will help save unnecessary operations and biopsies (Afza et al., 2022; Khan et al., 2021b).

For the multiple classifications of skin cancer, a new automatic approach named MSCDNet is proposed using dermoscopic images in this article. For skin cancer classifying and segmenting, the developed approach provides exceptionally high accuracy based on the DenseUNet and SqueezeNet methodologies. Dermoscopic images are used in this research to classify skin cancer automatically (Demir, Yilmaz & Kose, 2019). Early skin cancer detection provides a more significant opportunity for starting treatment on time and halting the disease’s growth for medical professionals. Also, the effectiveness of MSCDNet is evaluated compared to the most advanced medical deep learning models, such as Mask RCNN-DenseNet201, ConvNet, ARL-CNN, Inceptionv3, and CSLNet (Gonzalez-Diaz, 2018). The multiple skin cancer classifications are classified in this research using dermoscopy images, including Benign Keratosis, Melanoma, Melanocytic Nevi, and Basal Cell Carcinoma.

The main contributions of the research are:

• Pre-processing is the first stage; we perform hair removal and noise elimination in the pre-processing stage. From the dermoscopic images, the Laplacian-based algorithm removes hair, and the median filter is employed to eliminate the noise.
• After the completion of a pre-processing phase, the features are selected with the help of the BDA algorithm. Then for effective classification, the SqueezeNet deep learning model is used. After the classification of multiple skin cancers, skin lesion area recognition and segmentation are done using the DenseUNet model.
• The ISIC 2019 dataset images are used to train the proposed MSCDNet model, containing dermoscopic images of basal cell carcinoma, melanocytic nevi, benign keratosis, and melanoma. A 70:20:10 ratio of images is used for training, testing, and validating the proposed model. The implementation is done by using the Python platform.

The remaining article structure is described as follows. ‘Proposed Methodology’ discusses the literature review. The proposed methodology is described in ‘Methodology’. ‘Result and Discussions’ explains and discusses the experiments conducted, and ‘Conclusion’ presents the conclusion.

In the early detection of the disease, research on skin cancer diagnosis has been extensively explored to assist medical professionals. Recent research, however, focuses on employing various artificial intelligence algorithms to detect different types of skin malignancies automatically.

A new deep learning model is developed by Khan et al. (2021b) for classification and segmentation. The skin lesion classification is performed by using DCNN, and skin lesion segmentation is done by optimized color feature (OCF). The artifacts are removed, and lesion contrast is enhanced by proposing a hybrid technique in this research. Then the OCF is used for color segmentation. An existing saliency approach is used for the enhancement of the OCF approach, and a new pixel-based method is used for fusing the approach. A new parallel fusion technique is used to create a DCNN-9 model for extracting the deep features and fusing these features with the OCF approach (Saeed & Zeebaree, 2021).

The automated hyperparameter optimized CNN was developed by Mohakud & Dash (2022) for determining the skin cancer classification in this research. The hyperparameters of CNN are optimized using the Grey Wolf Optimization algorithm applying the appropriate encoding technique. The hyperparameter optimization based Genetic algorithm and particle swarm optimization are compared by determining the model performance using a multi-class skin lesion dataset.

For skin cancer classification, Aldhyani et al. (2022) proposed a simple and effective model. The developed approach accurately classifies the skin cancer images with the practical usage of activation functions and kernels. The most excellent classification results are achieved by layering dynamic-sized kernels, which provide only a few parameters.

A novel multi-class skin lesion classification model is proposed by Arshad et al. (2021) in this research. There are several steps in the developed framework. Augmentation is done in the first stage. Up and down flip, right-left flip, and rotate 90 operations are performed for augmentation. Deep models are fine-tuned in the second step. ResNet-101 and ResNet-50 models are selected, and their layers have been updated. On augmented datasets, both fine-tuned deep models are then trained by applying transfer learning. A modified serial-based method is used to extract and reuse valuable features in the succeeding stage.

A novel CNN model is developed by Iqbal et al. (2021) for skin cancer diagnosis. An advanced deep learning model is designed, implemented, and validated in this research for multi-class skin lesion classification. The performance of the proposed model is increased effectively by designing the DCNN model with lower parameters and filters, multiple filter sizes, and several layers.

A weighted average ensemble learning-based model is proposed by Rahman et al. (2021) for classifying multiple skin cancer classifications. The ensemble of DenseNet, Xception, ResNet, SeResNeXt, and ResNeXt are used for the classification. The grid search method was then used to identify the best base model ensemble combinations, and the effect of every base model was optimized for the average recall score.

A novel residual deep convolutional neural network (RDCNN) is proposed by Hosny & Kassem (2022) for diagnosing skin lesions. Using the segmented images, the suggested RDCNN is tested. Then, a new dataset is used to train the model.

A novel CNN approach is proposed by Hosny, Kassem & Foaud (2020). The suggested strategy includes three primary phases. For input images, the ROI is segmented in the initial pre-processing step. The segmented ROI images are improved by translation and rotation modifications. Several DCNN configurations are employed as a final step, including Alex-net, ResNet101, and GoogleNet.

A two-stream deep neural network information fusion framework is proposed by Khan et al. (2022a) for multi-class skin cancer classification. Two streams are followed by the developed approach. For contrast enhancement, a fusion-based technique is suggested first. This provides enhanced images to the pre-trained DenseNet201 architecture. A skewness-controlled moth–flame optimization algorithm is used for optimizing the extracted features. The proposed feature selection framework is used for extracting and downsampling reliable features. A new parallel multi-max coefficient correlation approach is utilized for the feature-combining process. Images of lesions are categorized using a multi-class extreme learning machine classifier (Thurnhofer-Hemsi & Domínguez, 2021).

For the precise identification of skin cancer, Femil & Jaya’s 2023 systematic image processing method was used. With the use of the Gabor filter, noise is removed from the dermoscopy images of skin lesions. A cascaded fuzzy c-means (FCM) algorithm is then used to segment the pre-processed dermoscopy image into different regions. The melanoma parameters are effectively extracted by using Gabor response co-occurrence matrix (GRCM). To significantly identify the features, The probabilistic neural network (PNN) classifier is used to categorize the phases of skin lesions best.

A CNN-based DSCC_Net was introduced by Tahir et al. (2023). The skin cancer disease’s visible characteristics of classification methods are shown using the Grad-CAM heat-map technology.

## Proposed Methodology

### Problem statement

Most of the prior deep learning models produce effective results. However, it has some limitations for effective classification, such as it requires numerous steps for analyzing the data, the retrieved handcrafted features cannot guarantee higher classification performance, and poor multi-class classification problems are attained due to an imbalance in data distribution. We propose a new automatic deep learning-based MSCDNet model for accurate classification and segmentation of multiple skin cancer classifications for solving this problem.

### Methodology

**Figure 1:**
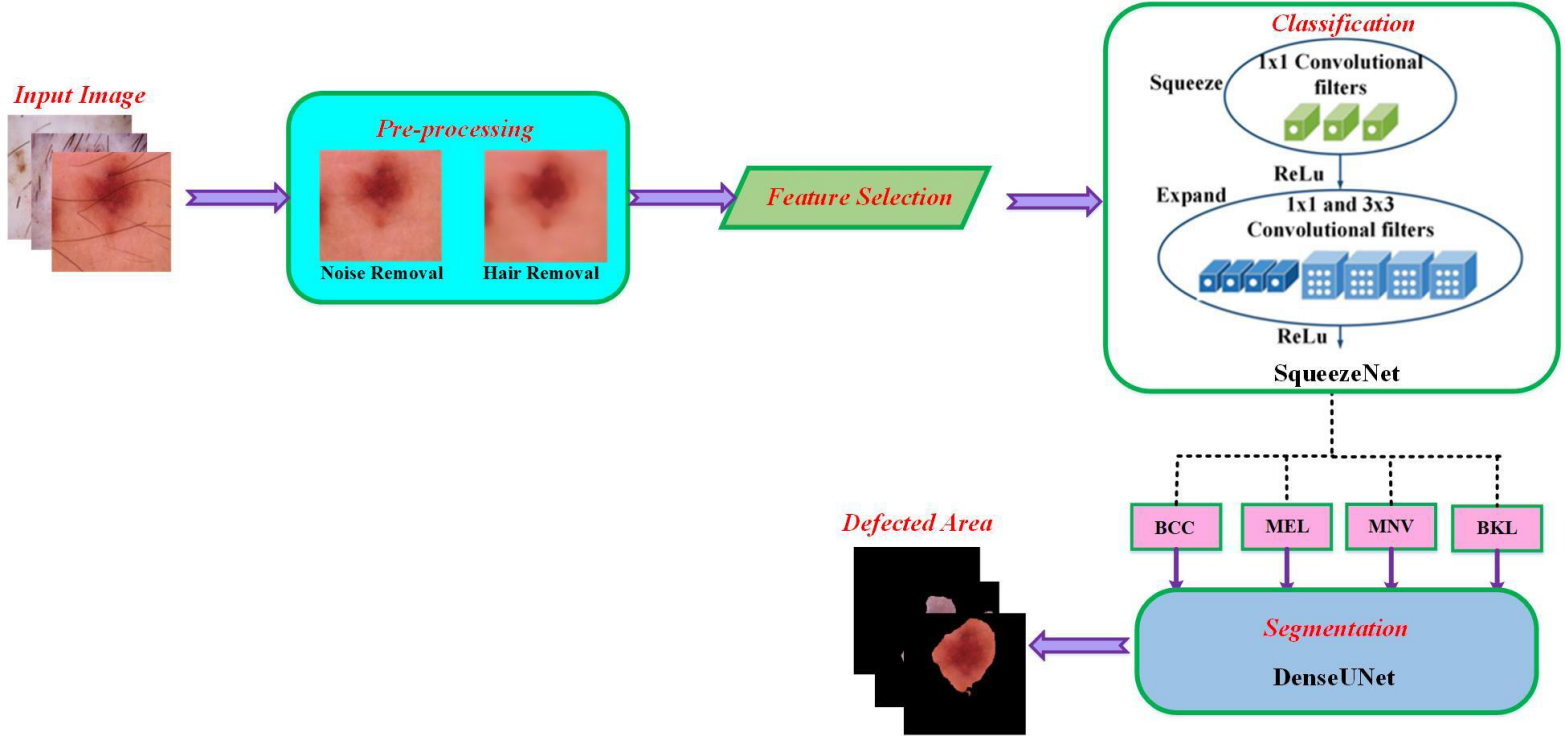
Schematic diagram of the proposed methodology.

For multiple skin cancer classification, a new MSCDNet deep learning model is proposed in this article. In the training of the proposed model, ISIC 2019 dataset is utilized. First, performs the pre-processing operation in the dataset images to match the input dimension of the proposed model. Input image sizes are fixed to 224 × 224 resolution for this reason. Additionally, the normalization process is helpful in the images for preventing the overfitting of the model. For detecting skin cancer disease, the proposed model has four primary stages: (1) Pre-processing, (2) selecting the features, (3) classification, (4) segmentation, and (5) performance evaluation. The pre-processing is performed by using a Laplacian-based algorithm and a median filter. Using a Laplacian-based algorithm, the hair is removed from dermoscopy images. Then using a median filter, the unwanted noise from the dermoscopic images is removed. Once the images were pre-processed, we fed them into the feature selection phase to decrease complexity time. With the help of the BDA algorithm, the selection of valuable features is done. The next phase is classification. The obtained features are sent into the deep learning classifier SqueezeNet for multiple skin cancer classifications. This classification network classifies the dermoscopy images into basal cell carcinoma (BCC), benign keratosis (BKL), melanoma (MEL), and melanocytic nevi (MNV). After classification, DenseUNet is employed to segment the defective area. The overall structure of the proposed MSCDNet model is shown in Fig. 1.

### Dataset description

**Figure 2:**
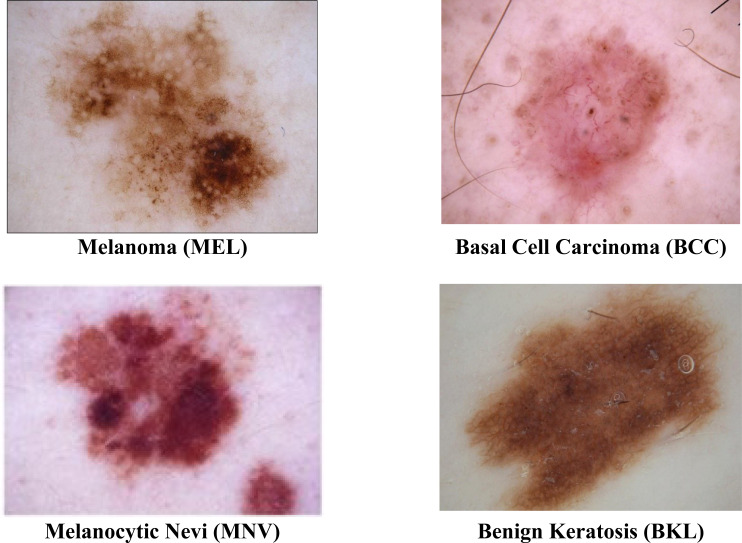
Sample images of multiple skin cancer classification.

The ISIC 2019 was the most often used dataset in this area of research. At https://challenge.isic-archive.com/data#2016, the dataset is obtained. Due to the uneven distribution of images among the eight groups in the ISIC 2019 dataset, it is among the most challenging datasets to classify. With multiple skin cancer classes, the dataset includes 25,331 dermoscopic images. A total of 2,624 benign keratosis images, 3,323 basal cell carcinoma images, 12,875 melanocytic nevi images, and 4,522 Melanoma images are present in the dataset. The rest of the other class images in the dataset are not considered. For training, testing, and validation sets, the dataset is divided into a ratio of 70%:20%:10. A description of the dataset is shown in Table 1. The sample images from the ISIC 2019 datasets are displayed in Fig. 2.

**Table 1:** Classification of datasets.

Dataset division	Benign Keratosis (BK)	Basal Cell Carcinoma (BCC)	Melanoma (MEL)	Melanocytic Nevi (NV)	Total images
Training set	1,837	2,327	3,166	9,013	17,731
Testing set	524	664	904	2,575	5,066
Validation set	263	332	452	1,287	2,533
Total	2,624	3,323	4,522	12,875	25,331

### Data augmentation

Deep learning models require a lot of data to function correctly and generalize. Data augmentation techniques include flipping, vertical and horizontal shifting, zooming, random shearing, aspect ratio, pixel jittering, adjusting contrast, modifying brightness, random cropping, flipping, and rotation. Instead of acquiring new data, intentionally increasing the quantity of data is known as “data augmentation.” which is already accessible by adding copies of training data that have been significantly altered. Oversampling or data warping is used to purposefully increase the training dataset’s size or prevent the model from over-fitting from the very start. By making slightly modified copies of previously existing data or by generating synthetic data from previously existing data, it is utilized to increase the diversity of the data.

Rich and poor sets were subtly separated from the ISIC 2019 dataset to improve it relatively. A total of 23,344 examples from the “BCC,” “BKL,” “MEL,” and “NV” cancer classes make up the rich set. The poor set, on the other hand, has 1987 instances of the “AK,” “DF,” “SCC,” and “VASC” classes. As a result, the rich set takes up 92.16% of the dataset, whereas the poor set only accounts for 7.84% of the dataset. To balance the training dataset’s imbalance, augmentation parameters should be applied to the poor but not the rich set classes. To save resources and to label time at the risk of lengthening training time, the online augmentation process is performed for a poor set augmentation process.

### Pre-processing

First, the input images are pre-processed using the Laplacian-based algorithm and the median filter. Before using the dermoscopic input images as input for segmentation, hair is first removed since it can significantly or partially conceal some types of melanoma. After converting the image to grayscale, the Laplace operator-based sharpening filter is employed. Then the filtered image is subtracted from the original image. By utilizing a median filter, unwanted image noise is removed. When it comes to keeping an image’s edges, the median filter performs admirably. When the hair has been removed, noise is removed from the images using a median filter.

**Figure 3:**
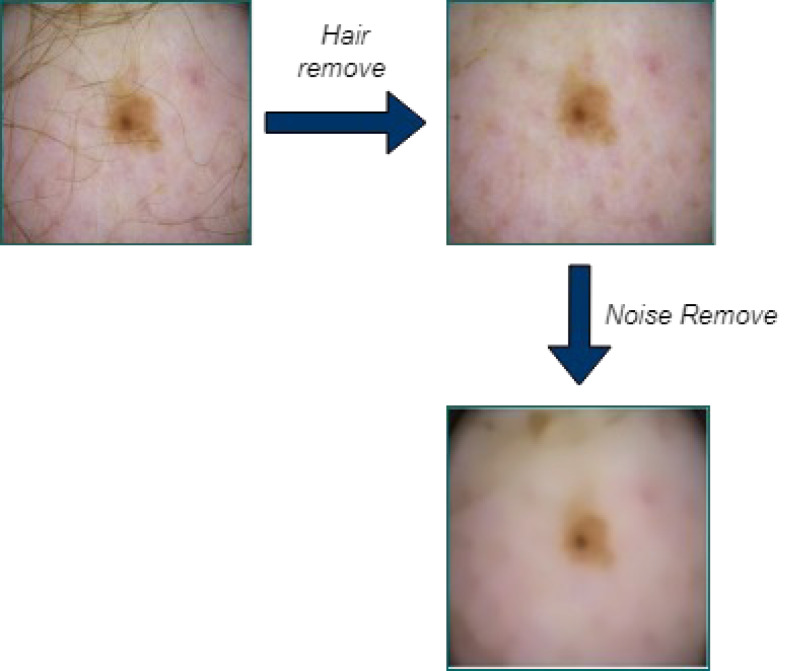
Structure of SqueezeNet model.

In the dataset, the dermoscopy images are characterized by 450 × 600-pixel resolution. We reduced the image resolution to 224 × 224 pixels to standardize them with the model input. Additionally, the proposed approach’s proper training was ensured by using the data normalization technique. We then prepared the datasets for MSCDNet input for training. During the pre-processing phase, Fig. 3 shows how well the noise and hair removal procedures worked.

#### Laplacian filtering

A Laplacian filter is applied to the pre-processed image. This filter highlights areas of rapid intensity changes, including edges and hair regions.

#### Thresholding

A threshold is applied to the Laplacian image to create a binary mask. The threshold should be set to separate the hair regions from other structures. This step aims to generate a mask where hair pixels are assigned a value of 1 while non-hair pixels are assigned a value of 0.

#### Morphological operations

Morphological operations such as erosion and dilation are used to refine the binary mask. These operations can help remove small artifacts and smooth the hair regions while preserving the important structures in the image.

#### The hair removal process uses a sharpening filter based on the Laplace operator

In our proposed research, a sharpening filter-based Laplacian operator is used to remove the processed hair. An example of a second-order or second-derivative enhancement method is the Laplacian of a picture, which draws attention to areas of rapid intensity variation. It is an incredibly effective derivative method of improvement. It excels in locating the fine details in an image. An operator with the Laplacian function will improve any feature with a strong discontinuity. It is commonly known that the Laplacian is a linear differential operator that approximates the second derivative provided by Eq,

\begin{eqnarray*}{\nabla }^{2}f= \frac{{\partial }^{2}f}{\partial {x}^{2}} + \frac{{\partial }^{2}f}{\partial {y}^{2}} \end{eqnarray*}

where *f* denotes the image.

### Noise removal process using the median filter

Due to the nonlinear nature of the median filter, the mathematical analysis of the image with random noise is rather challenging. The average filtering’s noise variance is

\begin{eqnarray*}{\sigma }_{0}^{2}= \frac{1}{n} {\sigma }_{i}^{2} \end{eqnarray*}

where input noise power is represented as ${\sigma }_{i}^{2}$ the size of the median filtering mask size is represented as n, the noise density function is depicted as $f(\overline{n})$. The effects of median filtering depend on two factors, noise distribution, and mask size. Random noise reduction’s median filtering performance outperforms the average filtering performance. It is quite efficient to use the median filter.

### Feature selection

The feature selection process before the use of classification algorithms is important since it improves the classifier’s overall performance, decreases the probability of algorithm failure, and reduces algorithmic computing complexity. Effective classification results are typically achieved by combining multiple features. To improve a classification model’s capacity for discrimination, feature selection is required. For the feature selection process, the redundant or irrelevant features are reduced by discovering the most significant subset of the initial feature set.

This work aims to solve the problem of feature selection by using an algorithm to select features. To accomplish this, the BDA is the primary method used. In 2016, Mirjalili presented the BDA; DA’s discrete variation is called BDA. The relations of dragonflies in identifying the food source (the best solution) and avoiding the enemy (the worst solution) serve as a model for the exploitative and exploratory DA processes. Updating a position in BDA uses five significant behaviors: distraction, attraction, cohesion, alignment, and separation.

The following are descriptions of each of these behaviors.

The static collision between the present individual and a nearby individual is prevented by the Separation process. Separation can be described mathematically as follows:

(1) \begin{eqnarray*}S{N}_{i}=-\sum _{j=1}^{N}Q-{Q}_{j}\end{eqnarray*}

where the number of neighbor individuals is represented by *N*, a dragonfly’s position is indicated by *Q*, and the neighbor individual’s position is described as *Q*_*j*_

A swarm or sub-swarm of individuals can match their velocity because of the alignment process. The alignment is determined as follows:

(2) \begin{eqnarray*}AG{N}_{i}= \frac{\sum _{j=1}^{N}{V}_{j}}{N} .\end{eqnarray*}

The velocity of neighbor individuals is represented by *V*_*j*_, and the number of neighbor individuals is represented by *N*.

Cohesion is the current individual’s divergence from the mass of nearby individuals toward its center. The cohesion is defined as follows:

(3) \begin{eqnarray*}CH{N}_{i}= \frac{\sum _{j=1}^{N}{Q}_{j}}{N} -Q\end{eqnarray*}

where *j*-th dragonfly’s position is indicated by *Q*_*j*_, and the overall neighborhood dragonfly number is described as *N*.

Individuals in swarms in nature draw toward food sources and divert predators in addition to separating, aligning, and forming a cohesive group.

According to attraction, the individual must be attracted to possible food sources. The attraction is denoted mathematically as:

(4) \begin{eqnarray*}{F}_{i}=Qf-Q\end{eqnarray*}

where the position of a food source is indicated by *Qf*

The individual should be kept away from a potential predator is implied as a distraction. Calculations for the distraction are as follows,

(5) \begin{eqnarray*}D{T}_{i}=Qe+Q\end{eqnarray*}

where the position of the enemy is depicted by *Qe*

These five behaviors in DA control the movement of dragonflies. The following step vector is calculated for updating the position of each dragonfly:

(6) \begin{eqnarray*}\Delta {Q}_{i}(t+1)=(sS{N}_{i}+aAG{N}_{i}+cCH{N}_{i}+f{F}_{i}+eD{T}_{i})+w\Delta {Q}_{i}(t+1)\end{eqnarray*}

where the separation weight, alignment weight, cohesion weight, food weight, predator weight, and inertia weight are represented as *s*, *a*, *c*, *f*, *e*, *w,* and the current iteration is represented by *t*.

The following equation is used to update the dragonfly positions in the original DA:

(7) \begin{eqnarray*}{Q}_{i}(t+1)={Q}_{i}(t)+\Delta Q(t+1).\end{eqnarray*}

This method can handle significant challenges because of such movement and navigation. The following equations are used to update the position vectors in BDA, unlike DA:

(8) \begin{eqnarray*}{Q}_{i}^{d}(t+1)& = \left\{ \begin{array}{@{}l@{}} \displaystyle 1-{Q}_{i}^{d}(t)rand\lt TF(\Delta {X}_{i}^{d}(t+1))\\ \displaystyle {Q}_{i}^{d}(t)rand\geq TF(\Delta {X}_{i}^{d}(t+1)) \end{array} \right. \end{eqnarray*}

(9) \begin{eqnarray*}TF(\Delta Q)& = \left\vert \frac{\Delta Q}{\sqrt{\Delta {Q}^{2}+1}} \right\vert \end{eqnarray*}

where ${Q}_{i}^{d}$ is the *i*-th dragonfly’s position in the *d*-th dimension, the step vector is represented as ΔQ, the transfer function is *TF* (.), and the randomly generated number is described as *rand* in the interval of 0 and 1. The current iteration is represented as *t*, as shown in Eq. (9).

During the optimizations, several types of local and global searches are provided by BDA with cohesion, alignment, and separation. The other elements that enable the dragonflies to take advantage of the best solutions and avoid the wrong solutions are attraction and distraction. The BDA algorithm is superior due to these five swarming behaviors.

The changing probabilities for the position of dragonflies are determined by the V-shape transfer function in the BDA algorithm. In contrast to previous binary metaheuristics, BDA allows the dragonflies to select values other than 1 and 0 using this transfer function. To obtain promising search space, BDA has a high exploration capacity. The reliable features of skin dermoscopy images are effectively selected by BDA based on the explanations given above.

### Classification

**Figure 4:**
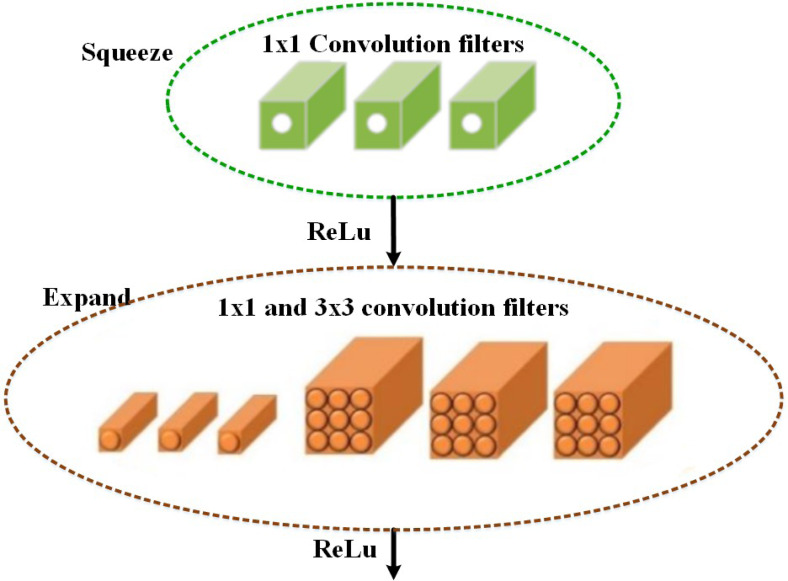
Schematic diagram of proposed Dense-UNet segmentation model.

Based on the selected features, the multi-class classification algorithm is used in the proposed MSCDNet model for effective image classification. The multi-class skin cancer classification uses a deep learning-based SqueezeNet model. During optimization, the SqueezeNet convolutional network is used. A test set is an input used in testing. The training and optimization stages for classification are done by the SqueezeNet convolution network. The layers of the fire modules are squeezed and expanded to create a simpler and more effective CNN model. The classification performance of the infection classes is determined by this most effective network model. The SqueezeNet model structure is given in Fig. 4.

 The fifteen layers are presented in the SqueezeNet structure, including one global average pooling layer, three max-pooling layers, two convolution layers, one softmax output layer, and eight fire layers. K × K notation denotes the filter’s receptive field size, the feature map length is represented by l, and the stride size is denoted by s. The features of the input to the network are RGB channels, and the input image size is 227 × 227. The convolution is used for normalizing the input images and applying the maximum pooling to the input images. In the input volumes, the convolution layer uses 3 × 3 kernels to convene small regions and weights.

An element-wise activation function is performed by each convolution layer, which is the conclusive part of its argument. SqueezeNet, consisting of squeezing and expansion stages, uses fire in its convolution layers. An identical input and output tensor scale exist for the fire. For classification, the 1 × 1 size filter is utilized by the squeeze phase, whereas the expansion phase uses the 3 × 3 and 1 × 1 size filters. The squeeze initially transmits the H × W × C input tensor. Also, the C/4 of the input tensor channels is equal to the number of convolutions. The data depth is increased by C/2 of the output tensor depth through the expansions following the initial phase of transmission of the information. The ReLu units are employed throughout the extension and squeezing processes. When keeping the feature’s size the same, the expansion and squeeze operations compress and expand the depth. Concatenate action is used for organizing the expansion outputs in the depth dimension of the input tensor. The feature maps are described as FM and channels are represented as C, a layer of output from the squeeze process is described as f{y}, and the kernel can be used to represent w, *i.e.,*

(10) \begin{eqnarray*}f\{ y\} =\sum _{fm1=1}^{FM}\sum _{c=1}^{c}{w}_{c}^{f}{x}_{c}^{fm1}\end{eqnarray*}

Here, *f*{*y*} ∈ *R*^*N*^ and *w* ∈ *R*^*c*×1×*FM*2^. Using the squeeze outputs, a weighted combination of several feature maps of a tensor is established. A downsampling operation in the spatial dimensions is performed by a downsampling operation in the network. The network uses an average global pool for creating a single value from the class maps with features. At the network’s result, multi-class probability distributions are created by utilizing the softmax activation function.

### Segmentation

**Figure 5:**
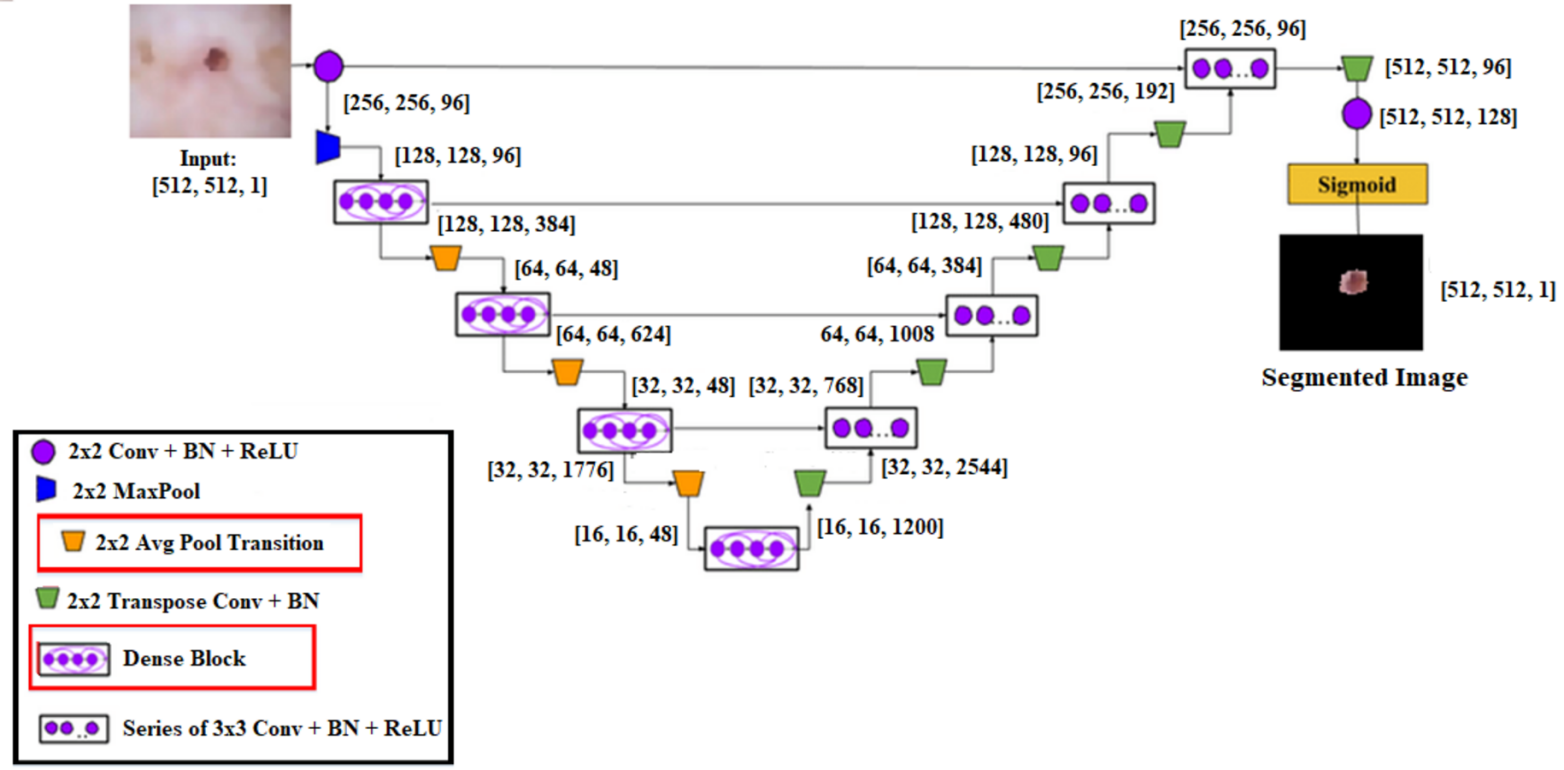
Experimental results of the proposed model.

A cascaded DenseUnet network model is developed to segment skin lesions precisely. For semantic segmentation, we carefully add the UNet model to the densenet model. The structure of DenseUnet is shown in Fig. 5. The down-sampling and up-sampling paths are used by this segmentation network for implementing the design concept of encoder–decoder of u-net and FCN. The continuous convolution operations are used for high-level and low-level semantic information extraction for the input images through the downsampling path.

 Until the complete resolution of the input images is recovered, the resolution of the feature map is expanded to the upsampling path through deconvolution. There are four transition layers and four dense blocks in the upsampling and downsampling paths. n consecutive convolution layers of the exact resolution are used to build the dense block. Each layer builds the dense block using a dropout layer, rectified linear units (ReLU), and batch normalization (BN).

The output feature maps are obtained by each layer’s input, specifically from all of its previous layers, which is called dense concatenation. The dense blocks must have the same feature size in each layer’s feature maps for the dense concatenation operation. There are 10 dense blocks in the proposed Dense-UNet model, such as five dense blocks in the dense upsampling path and five dense blocks in the dense downsampling path. Four densely connected layers are used for forming each dense block; each layer has the same feature size.

Layer transition is achieved by providing the transition block. The dense downsampling path consists of five layers, each of which is made up of a transition block and a dense block. In the dense upsampling path, the high-resolution images are reconstructed using dense block operation, merger operation, and upsampling layer. The five layers are used for making the path. The regions are localized, and the complete input resolution is recovered by this path.

In a convolutional neural network, the *l*th layer output is described by *x*_*l*_, and from the output of the preceding layer, a transformation *Q*_*l*_(*x*) can be used to calculate *x*_*l*_ *x*_*l*−1_ as:

(11) \begin{eqnarray*}{x}_{l}={Q}_{l}({x}_{l-1})\end{eqnarray*}

where *Q*_*l*_(*x*) is a sequence of operations, where the sequence of operations is depicted as *Q*_*l*_(*x*) convolution, pooling, rectified linear unit (ReLU), batch normalization (BN). From the previous layer, the skip connection output *Q*_*l*_(*x*) with the feature maps is integrated to improve the flow of information and enhance the training against vanishing gradients:

(12) \begin{eqnarray*}{x}_{l}=Q({x}_{l-1})+{x}_{l-1}.\end{eqnarray*}

However, the summation function is used for combining the output of *Q*_*l*_ an identity function, which can obstruct the flow of information in the network.

The skip connection is extended by introducing the dense connection to increase the information flow in the network. *x*_*l*_ isparticularly specified as:

(13) \begin{eqnarray*}{x}_{l}={Q}_{l} \left( \left[ {x}_{l-1},{x}_{l-2},\ldots ,{x}_{0} \right] \right) \end{eqnarray*}

**Figure 6:**
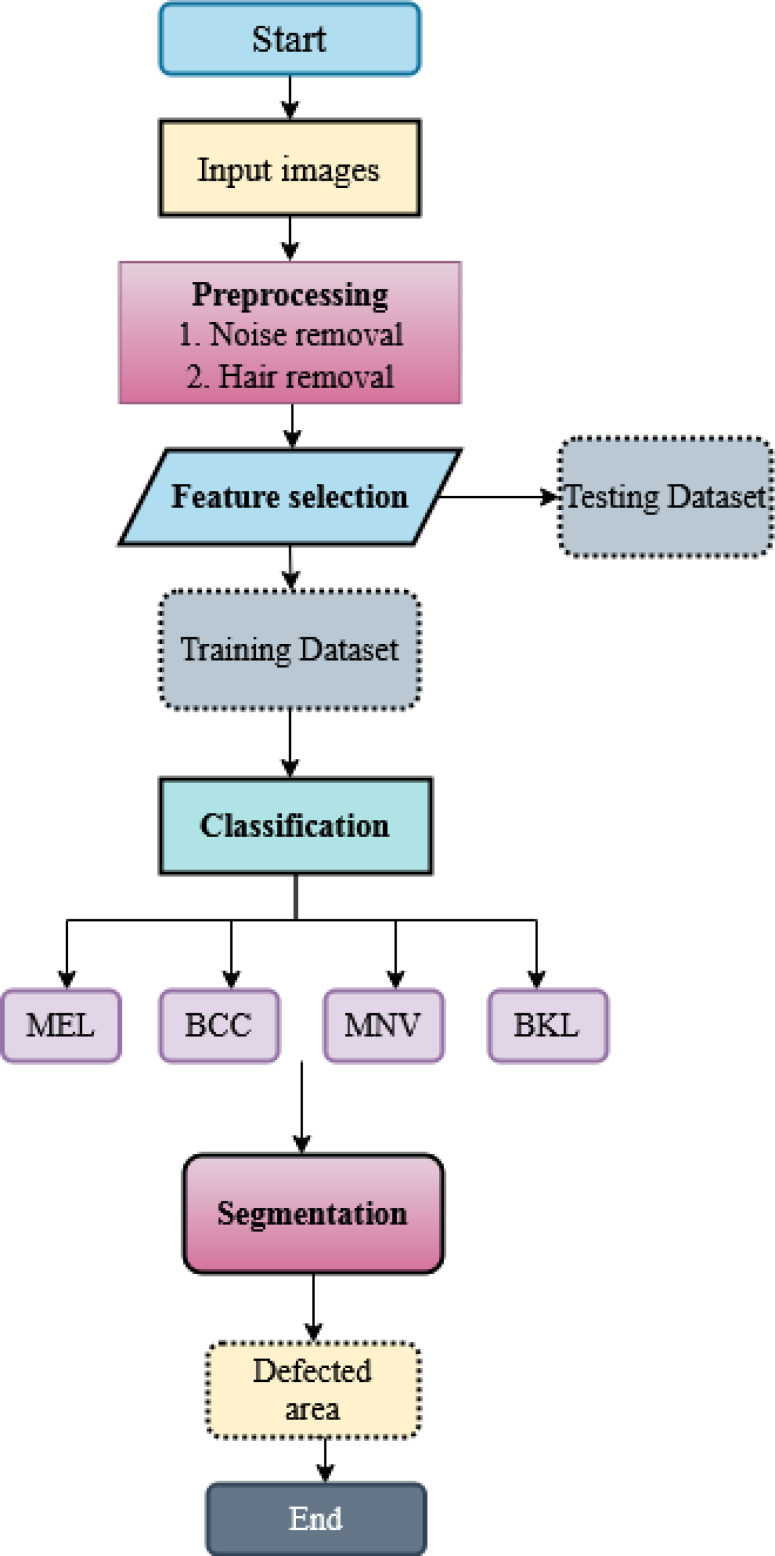
Proposed model’s ROC-AUC score.

Where the concatenation operation is represented by […]. A nonlinear transformation function is represented by H, which consists of dropout, Relu, BN, and convolution in this case. A transition layer connects the two dense blocks. A subsequent 2 * 2 average pooling layer, 1 * 1 convolutional layer, and BN layer are used to form the transition layer in the segmentation network. The flowchart of the proposed algorithm is shown in Fig. 6.

## Result and Discussions

This section covers the many performance metrics utilized to assess the proposed methodology and performance analysis of the proposed approach.

This research article was intended to classify different skin cancer diseases correctly. The ISIC 2019 dataset was classified and segmented using the proposed MSCDNet model. For training, testing, and validation sets, the dataset is divided into a ratio of 70%:20%:10. Multiple classifications of skin cancer diseases are classified by using SqueezeNet. The cascaded DenseUnet model is developed to segment multiple skin cancer diseases.

The proposed MSCDNet model uses dermoscopic images for classifying multiple skin cancer. The hyper-parameters of the proposed model are fine-tuned by using a “stochastic gradient descent” (SGD) optimization algorithm to improve the performance results. The number of epochs, batch size, and learning rate are optimized by the SGD algorithm. In 50 epochs, the proposed MSDNet training process is completed. The starting learning rate for the proposed MSCDNet is set to 0.05 by the SGD optimizer. The algorithm adjusts the momentum value is 0.8. After 10 epochs, the learning rate was reduced by 0.1 to avoid overfitting. The performance results of the proposed MSCDNet model are calculated regarding every class label by determining the recall, F1-score, precision, accuracy, confusion matrix, and ROC curve.

The proposed model is implemented by using the Keras library. Python is the programming language for implementing the proposed MSCDNet model.

### Evaluation measures

Resolved in this research are the problems with multi-classification. Melanoma, melanocytic nevi, benign keratosis, and basal cell carcinoma are accurately classified. Using a confusion matrix, an evaluation of the model’s effectiveness was conducted. F1-score, recall, precision, and accuracy are utilized for analyzing the proposed MSCDNet model performance.

The model is effectively classified by associating the model with a particular class, and the true positive (TP) indices comprise all data samples. In the confusion matrix, the true negative (TN) indices have additional samples. These samples are related to other successfully determined classes. The classifier’s estimated number of incorrect samples is referred to with the false negative (FN) and false positive (FP) indices in the uncertainty matrix. The performance metrics of the proposed model are calculated by using the following Eqs. (7)–(10).

In Eq. (1), accuracy is the proportion of true negative and true positive values divided by the confusion matrix’s total values. In the proposed model, the performance of the classifier is determined depending on the confusion matrix’s factors.

(14) \begin{eqnarray*}Recall& = \frac{TP}{TP+FN} \end{eqnarray*}

(15) \begin{eqnarray*}Precision& = \frac{TP}{TP+FP} \end{eqnarray*}

(16) \begin{eqnarray*}Accuracy(\%)& = \frac{TP+TN}{TP+FN+TN+FP} \end{eqnarray*}

(17) \begin{eqnarray*}F1-Score& = \frac{2(TP)}{2(TP)+FP+FN} \end{eqnarray*}

The computation of the evaluation metric is done by using ground truth labels and predicted labels.

### Experimental results

**Figure 7:**
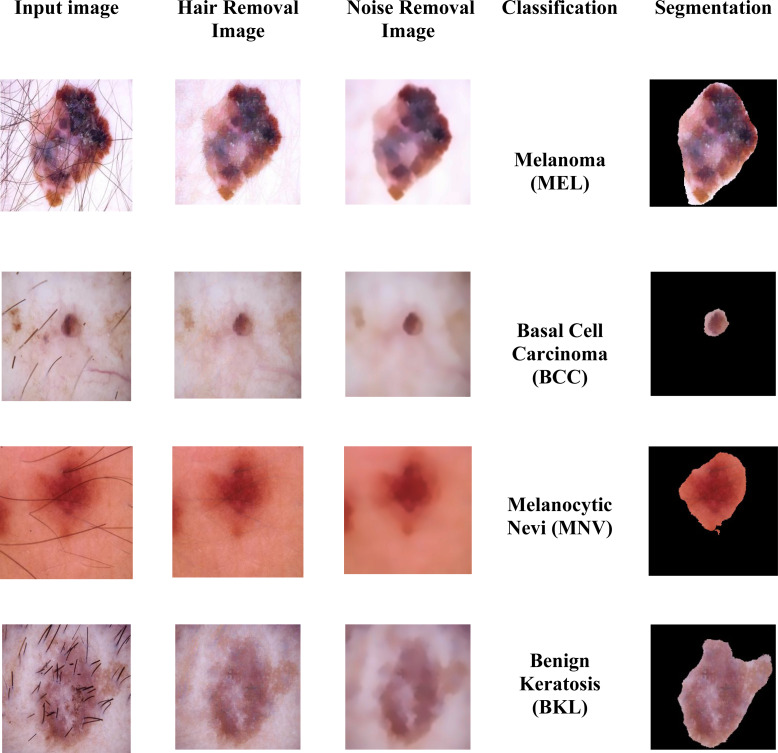
(A) Accuracy of the MSCDNet model for validation and training. (B) Loss of the MSCDNet model for validation and training.

The dataset images are divided into training, validation, and testing for experiment analysis. As training and validation sets, multiple types of malignant images are used for training the proposed MSCDNet model. A total of 50 epochs are used in the experiment. In training and validation, the proposed model achieved the predicted accuracy once all of the epochs were finished. The prominent features are combined and then classified images into the multi-classification of skin cancers by the proposed MSCDNet model for constructing the output images. For multiple classifications of skin cancer diseases, multiple performance measures are used for evaluating the proposed MSCDNet model. The dermoscopic images are used for assessing the proposed MSCDNet model’s classification accuracy. The training data of each class is used for training the proposed model. For testing the proposed model, available test data is used. The results of the suggested model’s experiments are provided in Fig. 7

The proposed model’s performance metrics are extracted by the confusion matrices, which are displayed in Table 2. The class-wise F1-score, recall, precision, and accuracy of the proposed model are shown in this table.

**Table 2:** Proposed model’s performance results for classification.

Class label	Accuracy (%)	F-score (%)	Recall (%)	Precision (%)
MEL	98.54	98.04	98.32	98.02
BCC	98.66	98.13	98.3	98.7
MNV	99.01	98.56	98.06	99.01
BKL	98.87	98.82	99.01	98.53

Melanoma (MEL) is classified with an accuracy of 98.54%, 98.87% for benign keratosis (BKL), 99.01% for melanocytic nevi (MNV), and 98.67% for basal cell carcinoma (BCC) classification. The performance results for the classification of the proposed model are effectively enhanced from the experimental results. The proposed model produced 98.77% accuracy, 98.76% F1-score, 98.42% recall, and 98.56% precision. The proposed model’s performance in terms of classes is determined by using a representative subset of the validation set’s images. The suggested deep learning model efficiently handles several skin cancer disorders of different classifications.

**Figure 8:**
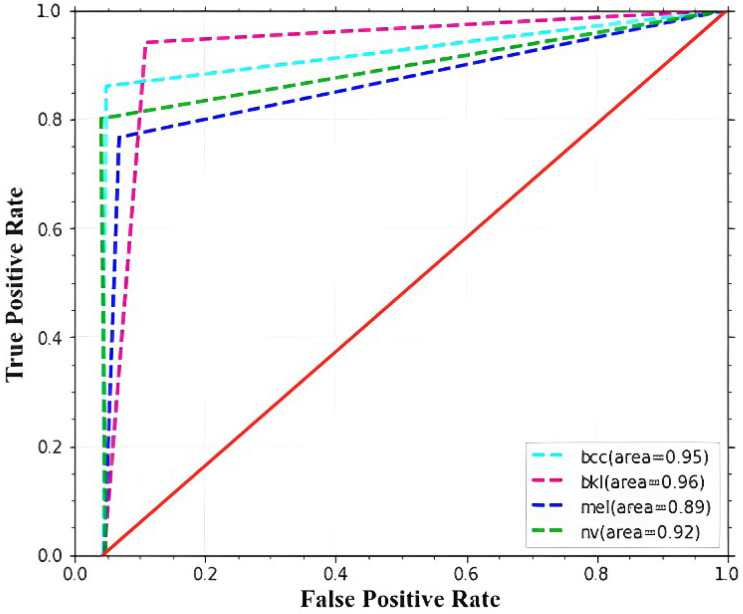
The confusion matrix of the proposed model.

The ROC curve and AUC score for each class are displayed in Fig. 8. All classes in the model have excellent AUC scores. If the model achieved the maximum AUC of the ROC, it is considered appropriate and effective. The ROC curve is calculated using the false-positive and true-positive rates. An AUC (ROC) of 0.9516 is attained by the proposed model.

**Figure 9:**
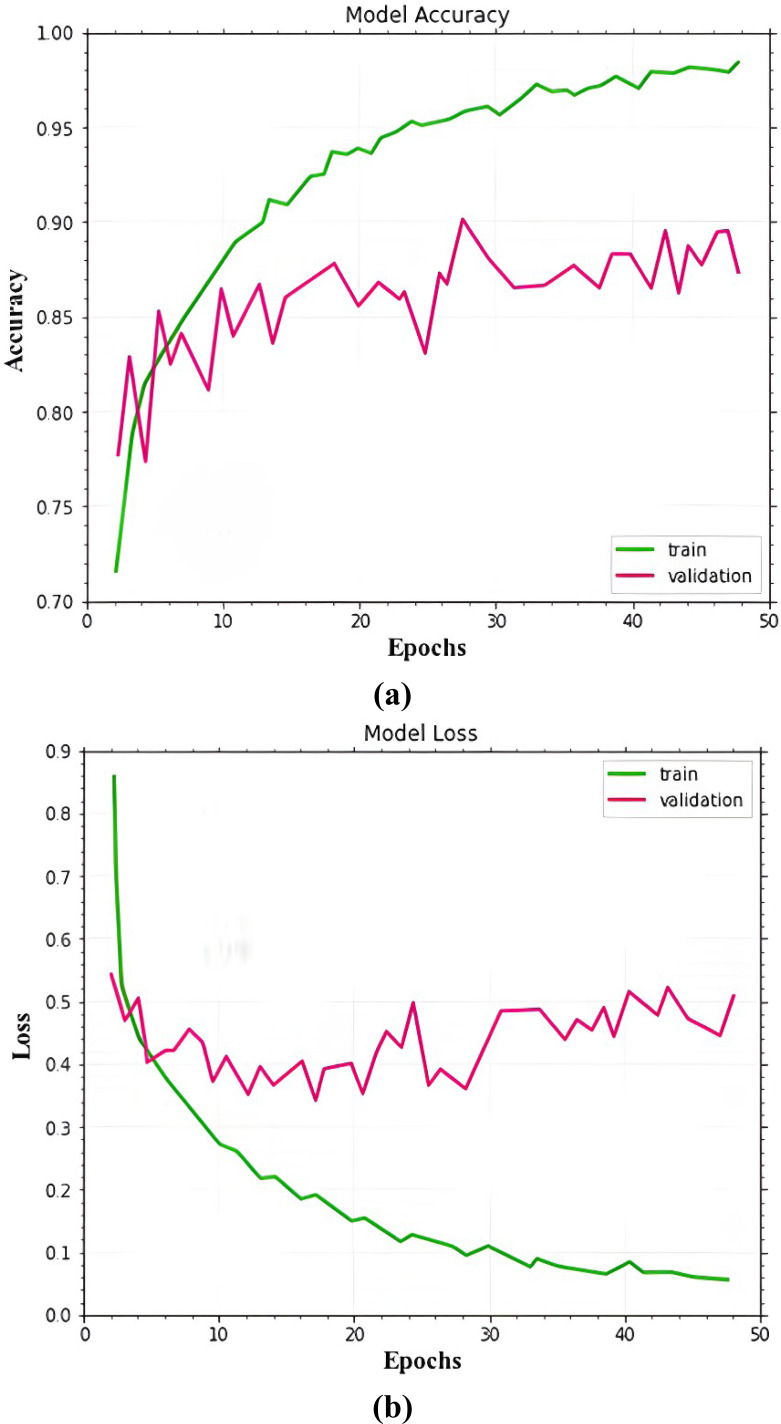
Comparative analysis of accuracy. (A) Model accuracy. (B) Model loss.

**Figure 10:**
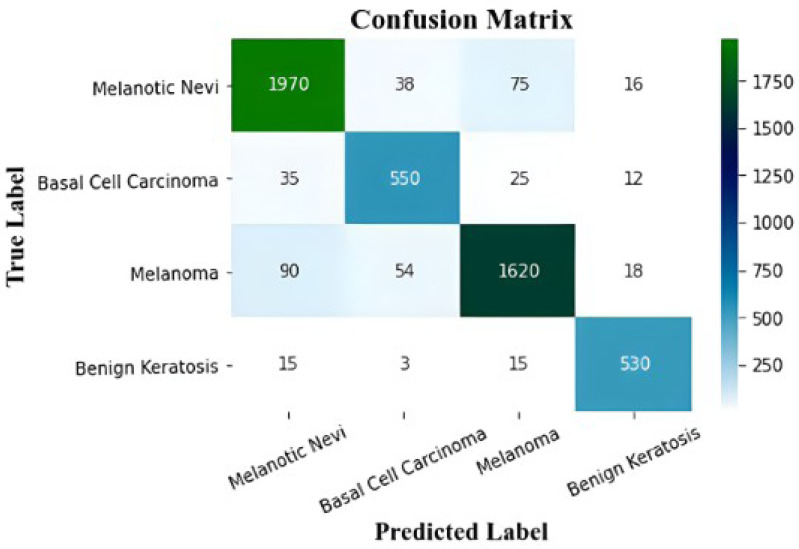
Performance comparison.

Figure 9 displays the MSCDNet validation and training accuracy throughout 50 epochs. A maximum of 98.77% accuracy is obtained for training, and a maximum of 92.15% accuracy is obtained for validation from the experimental results. Both training and validation achieve the highest level of performance. The model attains 0.011 for training and 0.069 for validation. The complete training process uses a timing of 1 min 54 s to arrive at the final iteration. The proposed MSCDNet is adequately trained and can correctly classify multiple skin malignancies. The proposed model’s confusion matrix is displayed in Fig. 10.

### Comparison results

The classification results of the proposed model with those of earlier studies are compared that used the same dataset in this section. With the most existing classifiers currently available, as listed in Table 3, the proposed MSCDNet is thoroughly analyzed regarding precision, f1 score, recall, and accuracy.

**Table 3:** Comparison of our method with other classification techniques.

Reference	Classification model	Recall (%)	Precision (%)	F1 score (%)	Accuracy (%)
Rahman et al. (2021)	Weighted Avg Ensemble	94	87	89	88
Mijwil (2021)	ConvNet	86.14	87.47	85.2	86.90
Khan et al. (2021a)	Mask RCNN-DenseNet201	–	95.00	94.90	96.3
Chaturvedi, Tembhurne & Diwan (2020)	Inceptionv3	84.00	83.00	84.00	92.83
Zhang et al. (2019)	ARL-CNN	87.80	86.70	–	86.80
Iqbal et al. (2021)	CSLNet	-	90.66	89.75	89.58
Zhao et al. (2021)	StyleGAN and DenseNet201	91.3	89.32	91.43	92.5
Gong, Yao & Lin (2020)	Inceptionv3 and StyleGANs	88	98.3	97.23	97.5
Kassem, Hosny & Fouad (2020)	Modified GoogleNet model	–	77	–	81
Proposed MSCDNet Model	SqueezeNet-DenseUNet	98.42	98.56	98.76	98.77

**Figure 11:**
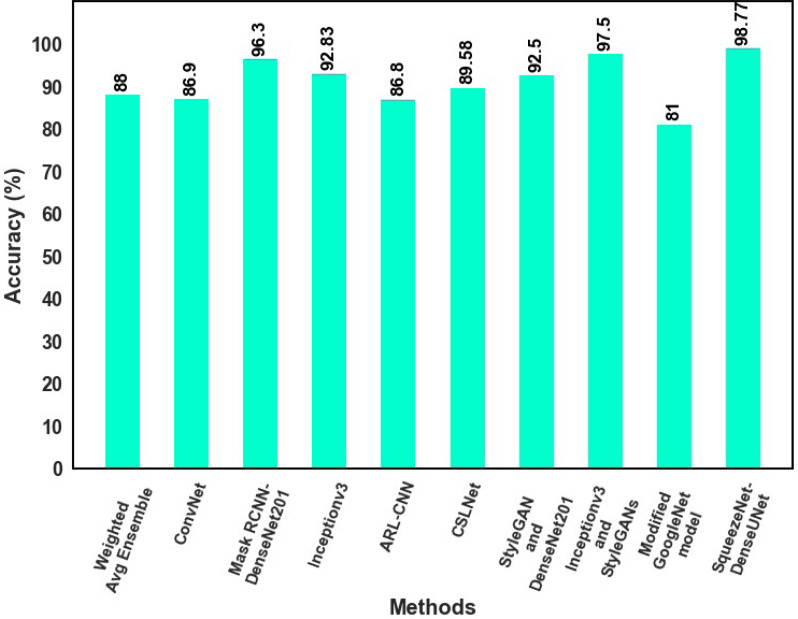
The comparative analysis of accuracy for the state-of-the-art model.

Inception v3-based Convnet net model for skin cancer diagnosis is provided by Mijwil (2021). The proposed model analyzes skin cancer into benign and malignant types. For multiple classifications of skin lesions, the Weighted Average Ensemble classifier is developed by Rahman et al. (2021), and this approach achieves an 88.00% of accuracy rate. 96.30% accuracy is achieved by Khan et al. (2021a) for multi-class skin cancer classification. A 92.83% accuracy rate for the multiple classifications of skin cancer is attained by Chaturvedi, Tembhurne & Diwan (2020). While comparing to Iqbal et al. (2021) and Zhang et al. (2019), 89.58% accuracy is achieved by Iqbal et al. (2021) and 86.80% accuracy is achieved by Zhang et al. (2019) for binary skin cancer classification. Kassem, Hosny & Fouad (2020) proposed a modified GoogleNet model for multi-skin lesions classification, achieving an 81% accuracy. The proposed MSCDNet classification model performs better when compared to the previous deep learning models and successfully detects the multi-class skin lesion classification. Due to the classification process, the overfitting and adverse effects on network performance are prevented by using the proposed classification network model. The comparative analysis of accuracy for the state-of-the-art model is shown in Fig. 11.

The inter-scale variability of the disease features is successfully extracted by using the proposed model. Additionally, the classification results of the proposed model are increased. The proposed method performs better than the existing methods for multiple skin cancer classification achieving 98.77% F1-score, 98.42% recall, 98.56% precision, and 98.77% accuracy. The suggested model’s impressive classification accuracy enables automated diagnosis and pre-screening of brain tumors.

## Discussion

Several skin malignancies are screened for and classified using dermoscopy images. To determine the disease and internally affected regions, the proposed model provides a comprehensive perspective of a specific region. For diagnosing multiple skin cancer disorders, dermoscopy is a more reliable and efficient technique. Since there are more and more confirmed cases of this deadly melanoma, a computerized diagnosis method is necessary. Using dermoscopy images, other skin cancer disorder patients and melanoma-positive patients are automatically discriminated against by employing deep learning models. Therefore, an MSCDNet model is proposed for effectively determining several skin lesion images and permits medical professionals to start these patients’ treatments earlier.

The infected areas are accurately classified by the proposed MSCDNet model from the experimental results. Also, multiple classes of skin cancer diseases are extensively trained by the proposed model. For the multi-classification of skin cancer, the proposed MSCDNet model performs better than the other recently existing classifiers. Furthermore, a 98.77% of accuracy rate is achieved by the proposed model for multiple skin cancer classifications. 224 × 224 × 3 resolution images are used to train the proposed model. While compared to the recent previous deep learning models, our proposed model gives an outstanding performance. It achieves 98.42% recall, 98.56% precision, 98.76% f1-score, 98.77% accuracy, and an AUC of 0.9516. The discriminative sequences and patterns of anomalies are efficiently identified by the proposed MSCDNet, which helps detect several types of skin cancer.

Additionally, we thoroughly explain why the proposed approach achieves higher performance at diagnosing diseases than previous approaches. In the existing deep learning models, fewer feature maps are provided by the final convolution layer’s spatial resolution, which limits the model’s ability to classify data accurately. An excessive number of input neurons that neglect crucial features and networks with incorrect filter sizes are further problems. The proposed MSCDNet model solves these problems. For effective skin cancer classification, a SqueezeNet-DenseUNet model with integrated expanded convolution values is used in this research. Also, the problem of overlapping in the diseased region and poor resolution of the dermoscopy image is solved by the proposed model. Furthermore, the convergence is accelerated by our proposed model while minimizing the effects of structured noise, leading to improved diagnostic performance. The proper filter size of 3 × 3 is used by the proposed model in the final step. For dermoscopy image-based multiple skin cancer classifications, the results of the experiments indicate that our proposed approach is a valuable medical tool for physicians.

## Conclusion

Even for professionals, manually identifying early-stage skin cancer from dermoscopic images is a difficult task, which has paved the way for its efficient automation. The traditional diagnostic techniques need to be updated due to the establishment of diagnostic systems based on deep learning. For multiple skin cancer diagnoses, a multi-classification MSCDNet is proposed in this research using dermoscopy images. In this research, multiple skin cancer diseases are classified and segmented using the proposed MSCDNet model. The dermoscopic images are first pre-processed to remove hair and noise. The valuable features are selected by using the BDA algorithm. After that, the multiple skin lesion classification is performed by using the SqueezeNet model. Finally, the DenseUnet model is used to segment the dermoscopy images.

Comparing MSCDNet to well-known deep learning-based networks, a comprehensive experiment reveals that it performs diagnostics with maximum efficiency. Based on the findings, the proposed MSCDNet model can be a valuable resource for medical professionals. The proposed model also increases the quality of the resulting image and makes it more convenient for the user, and it is an affordable method for skin cancer diagnosis. The association between skin burns caused by environmental factors like sunburn and other types of burns can be studied in a future study that builds on current work.

## Additional Information and Declarations

### Data Availability

The following information was supplied regarding data availability: Data are available at Figshare: Radhika, Vankayalapati; B. Sai Chandana, Dr (2023). MSCDNet-based Multi-class Classification of Skin Cancer using Dermoscopy Images. figshare. Dataset. https://doi.org/10.6084/m9.figshare.22547965.v1
